# Rutin as a Potential Therapeutic Agent for Multi-Organ Ischemia–Reperfusion Injury: From Multidimensional Mechanisms to Clinical Translation

**DOI:** 10.3390/molecules31071070

**Published:** 2026-03-24

**Authors:** Quan Peng, Yancheng Zhong, Xiaoxu Yang, Mei Yang, Xihua Cheng, Guozuo Wang

**Affiliations:** 1 Key Laboratory of Hunan Province for Integrated Traditional Chinese and Western Medicine on Prevention and Treatment of Cardio-Cerebral Diseases, College of Integrated Traditional Chinese Medicine and Western Medicine, Hunan University of Chinese Medicine, Changsha 410208, China; pengquan@stu.hnucm.edu.cn (Q.P.); zhongyancheng@hnucm.edu.cn (Y.Z.); yangxiaoxu@hunnu.edu.cn (X.Y.); 003779@hnucm.edu.cn (M.Y.); 2 Key Laboratory of Vascular Biology and Translational Medicine, Medical School, Hunan University of Chinese Medicine, Changsha 410208, China

**Keywords:** rutin, ischemia–reperfusion injury, oxidative stress, inflammation, cell death, signaling pathways

## Abstract

Ischemia–reperfusion injury (IRI) is a prevalent pathological process in clinical settings characterized by complex pathogenesis involving the interplay of oxidative stress, inflammation, mitochondrial dysfunction, and diverse cell death pathways. Fundamentally, IRI manifests as a complication arising from reperfusion therapies aimed at restoring blood flow following ischemia. Despite the existence of various therapeutic strategies, the development of effective interventions for IRI remains a significant challenge. Rutin, a low-molecular-weight flavonoid glycoside ubiquitously present in vegetables, fruits, and herbal medicines, exhibits promising therapeutic potential due to its pleiotropic biological activities, including antioxidant, anti-inflammatory, and cytoprotective effects against cell death. This review systematically elucidates the molecular mechanisms underlying the protective effects of rutin against IRI and synthesizes evidence from preclinical studies regarding its diverse modes of action. However, the clinical application of rutin is currently hampered by its relatively low bioavailability. Future research should prioritize the development of innovative pharmaceutical formulations to enhance its bioavailability, thereby fully unlocking its clinical translational value.

## 1. Introduction

Ischemia–reperfusion injury (IRI) is a pathophysiological process in which the restoration of blood supply to tissues or organs following a period of ischemia or hypoperfusion fails to recover function, and instead paradoxically exacerbates tissue injury, triggering severe inflammatory responses and dysfunction. Clinically ubiquitous and associated with high morbidity and mortality rates, IRI represents an inevitable secondary insult in various critical conditions, including recanalization for acute myocardial infarction, thrombolysis for ischemic stroke, organ transplantation (e.g., heart, liver, and kidney), major surgical procedures, and hemorrhagic shock [1,2]. The pathogenesis of IRI is multifactorial and complex, involving a burst of reactive oxygen species (ROS), mitochondrial dysfunction, inflammatory cytokine cascades, and various regulated cell death pathways such as apoptosis, pyroptosis, and ferroptosis. Collectively, these factors severely constrain the improvement of clinical prognosis in related major diseases [3]. Despite substantial advancements in modern reperfusion strategies—such as interventional therapies and thrombectomy—developing specific preventive and therapeutic measures targeting IRI itself remains a formidable challenge. Currently, there is a clinical paucity of targeted protective agents with defined efficacy and minimal side effects; existing interventions often yield limited outcomes and fail to effectively reverse microcirculatory disturbances and severe parenchymal cell injury. In this context, the identification of highly efficient and low-toxicity bioactive molecules from natural products has emerged as a pivotal direction in novel drug discovery. Rutin (also known as rutoside) is a natural flavonoid glycoside widely distributed in Sophora japonica, buckwheat, and various citrus fruits. In recent years, accumulating evidence indicates that rutin, owing to its superior antioxidant, anti-inflammatory, anti-apoptotic, and immunomodulatory properties [4], exhibits significant protective potential in IRI models across multiple organs—including the heart, brain, kidney, liver, intestine, and skeletal muscle. Consequently, it is increasingly recognized as a promising candidate agent for the prevention and treatment of reperfusion injury [1,5,6,7]. This review systematically summarizes research progress regarding the protective effects of rutin against IRI in various critical organs. Furthermore, it discusses current research limitations and future prospects for clinical application, aiming to provide novel insights for the clinical management of IRI.

## 2. Overview of the Biological Properties of Rutin

Rutin, a common secondary plant metabolite, appears as greenish-yellow needle-like crystals. It is variously known as rutoside, vitamin P, quercetin-3-O-rutinoside, or sophorin [8]. With a chemical formula of C_27_H_30_O_16_ and a molecular weight of 610.53, rutin belongs to the flavonol class and specifically constitutes the rutinoside (rhamnoglucoside) of quercetin. Structurally identified as 5,7,3′,4′-tetrahydroxyflavonol-3-rhamnoglucoside, it is hydrolyzed by gut microbiota into quercetin and subsequently metabolized into various downstream chemical products [9]. At room temperature, this compound manifests as yellow crystals with a melting point of approximately 178 °C. It possesses a bitter taste and is photosensitive, darkening upon exposure to light.

Similar to other flavonoids, rutin is widely distributed in various plant-based foods. Notably, the flower buds of Sophora japonica (Leguminosae), known as Huai Mi, contain up to 20% rutin [10]. It is also found in buckwheat, Ruta graveolens, jujube, hawthorn, ginkgo, wolfberry, and orange peel, as well as in medicinal herbs such as motherwort (Leonurus cardiaca), Bupleurum, Prunella vulgaris, aloe, and Gynostemma pentaphyllum [11]. As the human body cannot synthesize bioflavonoids, rutin must be obtained through dietary intake.

In animal models, rutin demonstrates a broad spectrum of physiological and pharmacological activities, including antioxidant [12], anti-aging [13], anti-inflammatory [14], neuroprotective [10,15,16], anti-diabetic [17], anti-cancer [18], anti-nociceptive [11,19], nephroprotective [20] and cardioprotective effects [12]. Furthermore, rutin delayed neurodegeneration in a rat model of Alzheimer’s disease [21].

**Figure 1:**
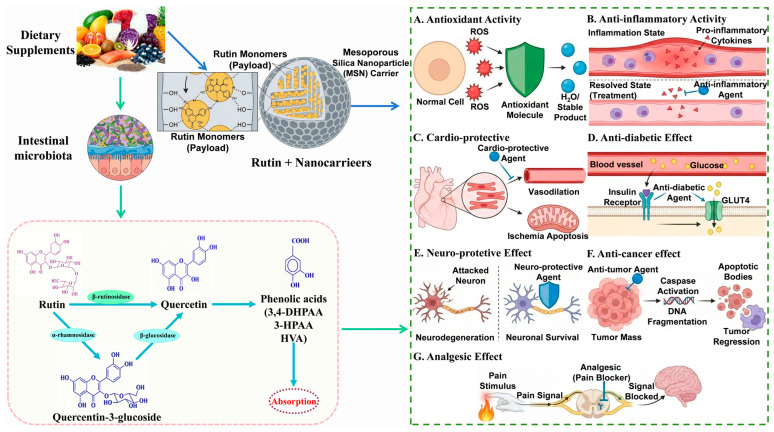
Schematic representation of the natural sources, intestinal absorption, and pharmacological activities of rutin.

Despite its therapeutic potential, flavonoids generally exhibit low bioavailability. Following oral administration, rutin is not efficiently digested by intestinal enzymes. Instead, gut microbiota-derived glycosidases—specifically α-L-rhamnosidase and β-glucosidase—hydrolyze it into its aglycone form, quercetin, which is then absorbed into the systemic circulation [22]. Consequently, the biomedical application of rutin faces significant hurdles, including low solubility, poor absorption, limited bioavailability, short half-life, and rapid metabolism [23]. In recent years, advancements in nanotechnology have offered promising solutions to these limitations. Accordingly, various nano-formulation strategies have emerged, aiming to enhance the solubility, absorption, and overall therapeutic efficacy of rutin. These strategies are primarily categorized into two classes: the direct preparation of rutin as nanocrystals without auxiliary compounds, and the construction of composite nano-systems by combining rutin with materials such as proteins, polysaccharides, lipids, polymers, or metals [24,25]. A schematic representation of the natural sources, intestinal absorption, and pharmacological activities of rutin is shown in Figure 1.

## 3. Pathophysiological Mechanisms of IRI

IRI is a complex pathophysiological phenomenon characterized by the paradoxical exacerbation of tissue damage following the restoration of blood flow and oxygen supply to ischemic tissues [26,27]. It is a primary cause of organ dysfunction and transplantation failure [26,28]. The underlying mechanisms involve a complex interplay at both cellular and molecular levels, including ROS generation, inflammatory responses, cell death, and calcium overload [27].

### 3.1. Oxidative Stress

Oxidative stress is defined by a disequilibrium between oxidant production and antioxidant capacity [29]. During IRI, abrupt alterations in the tissue metabolic milieu serve as a pivotal driver affecting oxidative stress and mitochondrial dysfunction.

#### 3.1.1. ROS

ROS are the primary mediators of damage in IRI, encompassing superoxide anions (O^2−^), hydroxyl radicals (OH^−^), and hydrogen peroxide (H_2_O_2_). In eukaryotic cells, approximately 95% of ROS originate from electron leakage at mitochondrial respiratory chain complexes [29]. In ischemic tissues, nitric oxide (NO) reacts with superoxide anions to produce highly toxic peroxynitrite [29]. During the ischemic phase, hypoxia impairs the mitochondrial electron transport chain (ETC), leading to the accumulation of tricarboxylic acid (TCA) cycle precursors such as NADH and succinate [30]. Concomitantly, cytochrome c (Cyt c) is lost during ischemia from the mitochondrion. CL is indispensable for the activity of mitochondrial complexes I, III, and IV [31]. Upon reoxygenation during reperfusion, although the ETC attempts to re-establish function, it remains compromised due to the depletion of CL and Cyt c. The rapid oxidation of accumulated succinate by succinate dehydrogenase (SDH) in early reperfusion initiates reverse electron transport (RET), driving electrons retrogradely from coenzyme Q (CoQ) to Complex I. This RET causes the over-reduction in Complex I and electron leakage to molecular oxygen, generating massive amounts of O^2−^ and H_2_O_2_, thereby triggering a ROS burst. Complex I is recognized as the most significant source of mitochondrial ROS generation during reperfusion [32]. ROS production is also mediated by xanthine oxidase (XO), which converts xanthine and hypoxanthine into uric acid while generating O^2−^ and H_2_O_2_. Under hypoxic conditions, NADPH oxidase (NOX) activity increases, utilizing oxygen to produce O^2−^ and H_2_O_2_. Furthermore, nitric oxide synthase (NOS) may become uncoupled due to the loss of tetrahydrobiopterin (BH4) during ischemia-hypoxia, leading to excessive ROS production [33]. Notably, clinical IRI may be driven by ‘reductive stress’ (i.e., NADH and NADPH accumulation) rather than traditional oxidative stress. This reductive stress is associated with the global activation of catabolic pathways (including glycolysis, fatty acid β-oxidation, autophagy, and glutaminolysis). These pathways generate excess reducing equivalents (NADH and FADH_2_) that exceed the oxidative capacity of the mitochondrial ETC or cytosolic lactate dehydrogenase (LDH), resulting in their accumulation [26].

#### 3.1.2. Mitochondrial Dysfunction

Mitochondria are the central hubs for oxidative metabolism and energy generation. Under hypoxic conditions, inhibition of the ETC causes a sharp reduction in ATP synthesis, which subsequently inactivates membrane electrolyte transporters and disrupts ionic homeostasis. Calcium overload activates xanthine oxidase, leading to a large production of ROS. The ensuing accumulation of ROS further damages the ETC, dissipating the mitochondrial membrane potential and exacerbating ATP deficits. In the context of renal IRI (RIRI), pathological outcomes are closely tied to dysregulated mitochondrial dynamics (fusion/fission), biogenesis, and autophagy [34]. IRI shifts the dynamic balance toward excessive fission—a process mediated by dynamin-related protein 1 (DRP1)—leading to mitochondrial fragmentation, ROS buildup, and cell death. While IRI suppresses the fusion proteins OPA1 and MFN2, targeting DRP1 inhibition has proven effective in attenuating RIRI [35]. Moreover, IRI compromises mitochondrial biogenesis by downregulating the key transcriptional regulator PGC−1α [36]. Regarding quality control, IRI triggers mitophagy to clear damaged mitochondria [37]. This process occurs via the ubiquitin-dependent PINK1-Parkin pathway or through ubiquitin-independent receptors such as BNIP3, NIX, and FUNDC1 that bind directly to LC3. Loss of PINK1, PARK2, or BNIP3 hinders this protective clearance and worsens renal injury. Similarly, in hepatic IRI, upregulation of PCSK9 exacerbates liver damage by inhibiting PINK1-Parkin-mediated mitophagy [34]. This inhibition promotes the release of mitochondrial DNA (mtDNA), thereby activating the cGAS-STING/NLRP3 inflammatory axis. Targeting PCSK9 blockade thus presents a viable strategy to mitigate mitochondrial damage [38].

#### 3.1.3. Imbalance of Antioxidant Defenses

Glutathione (GSH) is recognized as one of the most potent endogenous antioxidants within cells. In the context of hepatic IRI, the activity of γ-glutamyl transferase (γ-GT) is increased, driving the enhanced degradation of GSH. γ-GT hydrolyzes GSH into cysteinylglycine and glutamate. As a highly reactive thiol compound, cysteinylglycine initiates a redox cycle by reducing ferric iron (Fe^3+^) to ferrous iron (Fe^2+^). Subsequently, molecular oxygen oxidizes Fe^2+^ to generate O^2−^. This redox cycling results in elevated ROS levels, thereby exacerbating intracellular oxidative stress [39].

### 3.2. Inflammatory Response

Endothelial dysfunction driven by inflammasome activation, the release of pro-inflammatory cytokines and chemokines, and neutrophil infiltration constitutes a key pathophysiological mechanism of IRI. The initiation of this process is closely associated with mitochondrial injury, ROS accumulation, and pro-apoptotic signaling pathways [29].

#### 3.2.1. Inflammasome Activation

The activation of the NLRP3 inflammasome is critical to the pathology of IRI [40]. The assembly and activation of the inflammasome are triggered when injured cells release endogenous damage-associated molecular patterns (DAMPs). Subsequently, NLRP3 recruits ASC and procaspase-1 to form the inflammasome complex, thereby initiating the mechanism of pyroptosis [41]. The generation of ROS and reactive nitrogen species (RNS) further promotes tissue inflammation and the activation of the NLRP3 complex. Additionally, the leakage of mtDNA into the cytosol can activate both the cGAS-STING and NLRP3 pathways [42]. Moreover, ischemia-driven succinate accumulation acts as a distinct signal governing NLRP3 inflammasome activation [43].

#### 3.2.2. Release of Pro-Inflammatory Cytokines and Chemokines

The NF-κB signaling pathway acts as a central regulator of the inflammatory response in IRI, being universally activated across organs such as the heart, brain, liver, and kidney [29,44]. Once activated, NF-κB orchestrates the transcription of downstream genes and mediates the release of diverse pro-inflammatory cytokines. Crucially, NF-κB signaling functions as the essential priming step for NLRP3 inflammasome activation. Among the downstream effectors, TNF-α induces vascular endothelial injury and heightens capillary permeability, potentially compromising the integrity of the blood–brain barrier (BBB) [45]. Similarly, IL-6 is significantly elevated following renal IRI and propagates inflammatory signals via the IL-6/JAK/STAT3 axis [46]. IL-1β, predominantly derived from activated macrophages and monocytes, is a key mediator of sterile inflammation; it promotes leukocyte recruitment by upregulating endothelial adhesion molecules. Furthermore, macrophage-derived IL-1β engages STAT3 and NF-κB to drive the transcription of the angiogenic factor VEGF-A [47]. In contrast to these pro-inflammatory mediators, IL-10 functions as a protective anti-inflammatory cytokine. It suppresses macrophage activation and attenuates cytokine/chemokine production; consequently, IL-10 deficiency has been shown to aggravate AKI biomarkers, apoptosis, and inflammation in renal IRI models [48].

#### 3.2.3. Neutrophil Infiltration and Endothelial Dysfunction

In the early phase of reperfusion, neutrophil activation triggers the release of various products that act as chemokines, facilitating the recruitment of additional leukocytes. Following this, neutrophils secrete ROS and proteases, notably cathepsin G, which induces morphological changes in cardiomyocytes (CMs) and dismantles focal adhesions. In pulmonary IRI specifically, neutrophils further aggravate tissue damage via mechanisms such as the formation of neutrophil extracellular traps (NETs) [49].

Endothelial dysfunction plays a critical role in IRI, characterized by reduced nitric oxide (NO) generation and the upregulation of adhesion molecules [27]. This dysfunction promotes leukocyte adhesion, precipitating the ‘no-reflow phenomenon’ and microvascular obstruction (MVO). MVO is driven by vasoconstriction and microthrombosis. Additionally, NF-κB activation is implicated in endothelial dysfunction [50].

### 3.3. Modes of Cell Death

IRI culminates in cell death, encompassing a spectrum of programmed and non-programmed modalities such as apoptosis, necrosis, pyroptosis, and ferroptosis.

While necrosis is distinguished by cellular swelling and membrane rupture, apoptosis—a programmed process—is marked by nuclear shrinkage and chromatin condensation [51]. Pyroptosis represents an inflammatory form of regulated cell death involving membrane pore formation and the release of pro-inflammatory cytokines (IL-1β/IL-18). The NLRP3 inflammasome serves as the core machinery for pyroptosis; specifically, activated Caspase-1 cleaves Gasdermin D (GSDMD), or Caspase-3 cleaves GSDME, creating pores that result in cell lysis and inflammation [52]. Ferroptosis, an iron-dependent form of cell death, is defined by the toxic accumulation of lipid peroxides, primarily due to glutathione peroxidase 4 (GPX4) dysfunction. Morphologically, it presents with mitochondrial atrophy, increased membrane density, and cristae reduction. Mechanistically, Fe^2+^ drives lipid peroxidation by generating ROS through the Fenton reaction and serving as a cofactor for lipoxygenases (LOXs) [53]. In the context of hepatic IRI, the GLP-1 receptor agonist liraglutide and its metabolite GLP-1(9-37) have been shown to mitigate injury by suppressing ferroptosis. This protection is mediated by the reduction in lipid peroxidation via the GSK3β/Nrf2 pathway and the limitation of iron overload via the SMAD1/5/9/Hepcidin/FTH axis [54]. In renal IRI, ferroptosis involves the upregulation of ACSL4, which is subject to negative regulation by HIF-α [55].

### 3.4. Other Key Pathogenic Mechanisms

#### 3.4.1. Calcium Overload

IRI precipitates a disruption in calcium homeostasis, characterized by a pathological surge in intracellular calcium levels. Under hypoxic conditions, ATP depletion compromises the energy-dependent Na^+^/K^+^-ATPase, resulting in the retention of intracellular Na^+^. This ionic imbalance suppresses the activity of the Na^+^/H^+^ exchanger and the Na^+^/Ca^2+^ exchanger, thereby driving intracellular Ca^2+^ overload. This calcium excess exerts deleterious effects on mitochondria, manifesting as reduced ATP synthesis, dissipation of membrane potential, and the opening of the mitochondrial permeability transition pore (mPTP). Moreover, the accumulation of intracellular Ca^2+^ serves as a potent stimulus for oxidative stress and inflammasome activation [56].

#### 3.4.2. Metabolic Reprogramming and Lactylation

During ischemia–reperfusion (IR), the accumulation of L-lactate drives protein lactylation—a post-translational modification defined by the attachment of lactyl groups to lysine residues. In MIRI, there is a specific post-reperfusion surge in the expression of Serpina3k (SA3K) and its lactylation at the K351 site, a modification that confers enhanced stability to the SA3K protein. Predominantly secreted by cardiac fibroblasts (FBs), lactylated SA3K exerts a paracrine protective effect on CMs, shielding them from apoptosis. Mechanistically, this process recruits the Reperfusion Injury Salvage Kinase (RISK) and Survivor Activating Factor Enhancement (SAFE) pathways [57,58]. Conversely, in renal IRI, a deleterious mechanism is observed involving Hexokinase 2 (HK2), the rate-limiting enzyme of glycolysis. Histone lactylation at H3K18 transcriptionally activates the HK2 promoter, creating a positive feedback loop that intensifies renal injury [59].

#### 3.4.3. Gut Microbiota Dysbiosis and Intestinal Barrier Injury

In organ transplantation, IRI exerts a direct deleterious effect on the intestinal barrier, precipitating gut microbiota dysbiosis and heightening susceptibility to infection and rejection. Mechanistically, IRI triggers apoptosis in intestinal epithelial cells and downregulates key tight junction proteins such as Occludin and ZO-1. This disruption of barrier integrity enhances intestinal permeability, allowing for the translocation of toxins and microbes. Concurrently, IRI diminishes both IgA mRNA transcription and secretory IgA (sIgA) secretion, compromising mucosal immunity and facilitating bacterial translocation. In terms of the microbiome, IRI causes a significant reduction in microbial diversity, shifting the community profile towards dysbiosis. This is manifested by a decrease in beneficial genera (e.g., Bifidobacterium, Lactobacillus) alongside an overgrowth of opportunistic pathogens (e.g., Enterococcus, Enterobacteriaceae). This microbial imbalance ultimately results in reduced production of protective short-chain fatty acids (SCFAs) [28].

#### 3.4.4. Regulation by Non-Coding RNAs (ncRNAs)

ncRNAs are exemplified by long non-coding RNAs (lncRNAs), microRNAs (miRNAs), and circular RNAs (circRNAs). Recent evidence underscores the pivotal role of ncRNAs in modulating the pathogenesis of various organ disorders, including IRI [60]. Specifically, the circular RNA circMIRIAF is upregulated in myocardial IRI (MIRI) [61]. Acting as a competitive endogenous RNA (ceRNA), circMIRIAF sponges miR-544, resulting in the upregulation of WDR12. WDR12 subsequently activates the Notch1 signaling pathway, which intensifies oxidative stress and inflammation, ultimately aggravating MIRI.

**Figure 2:**
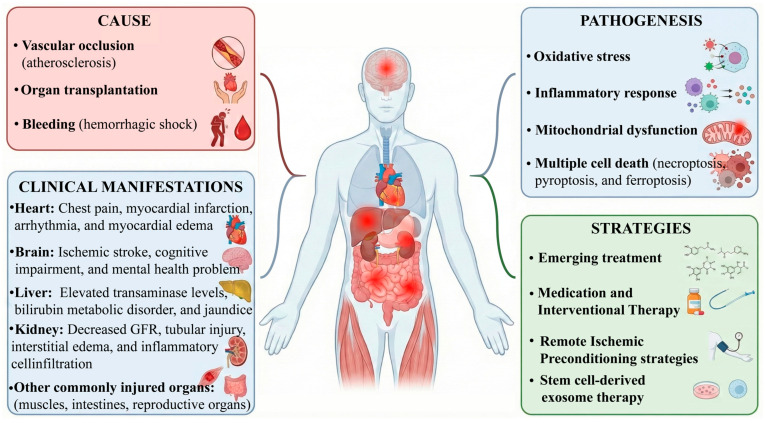
Cause, pathogenesis, clinical manifestations, and treatment strategies of IRI.

The pathophysiology of IRI is underpinned by a complex regulatory network involving multifaceted interactions and multiple signaling pathways. Contemporary research is moving beyond the traditional focus on early-stage oxidative damage and inflammation, delving into more refined molecular mechanisms such as regulated cell death, mitochondrial quality control, and gene expression networks. These insights provide a theoretical foundation for future precision medicine interventions. Additionally, novel nanoparticle strategies tailored to the properties of the BBB are paving the way for advanced therapeutic approaches in cerebral ischemia–reperfusion injury (CIRI) [62,63]. Cause, pathogenesis, clinical manifestations, and treatment strategies of IRI are shown in Figure 2.

## 4. Organ-Specific Protective Effects of Rutin Against IRI

### 4.1. Protective Effects of Rutin in CIRI

As shown in Table 1, research investigating Rutin and its complexes in the context of CIRI has consistently demonstrated clear neuroprotective efficacy. These benefits are orchestrated through a multifaceted approach involving antioxidant, anti-inflammatory, and anti-apoptotic activities, alongside BBB stabilization and the modulation of critical signaling cascades. The therapeutic potential of Rutin was highlighted as early as 1995 by Ortolani et al. in stroke patients [64]. By 2005, Lao et al. reported that Sophora japonica—a rich source of Rutin—reduced cerebral infarct volume and neurological deficits in rats, attributed to the suppression of microglial activation, IL-1β release, and apoptosis [64]. Subsequent investigations (2007–2009) centered on neuronal survival and cognitive preservation. Rutin was shown to reverse spatial memory impairment and inhibit neuronal death in the hippocampal CA1 region [65]. In a transient MCAO model, Rutin pretreatment bolstered antioxidant defenses, significantly elevating the activities of GPx, GR, CAT, GSH and SOD, while reducing levels of TBARS, H_2_O_2_, and protein carbonyls (PCs) compared to untreated controls [66]. Further exploration into mechanistic pathways revealed potent anti-inflammatory properties. For instance, Rutin was found to lower serum pro-inflammatory cytokines (TNF-α, IL-1β) and myeloperoxidase (MPO) in IRI models [67,68]. In a 2014 photothrombotic ischemia model, Rutin attenuated BBB disruption and improved neurological outcomes by suppressing matrix metalloproteinase-9 (MMP-9) [69]. Similarly, in a 2016 subarachnoid hemorrhage (SAH) model, Rutin mitigated brain edema and BBB damage, potentially by inhibiting the RAGE-NF-κB axis [70]. Similarly, Troxerutin and Troxerutin-Cerebroprotein Hydrolysate Injections (TCHIs) have been found to maintain BBB integrity and mitigate CIRI through antioxidant, anti-inflammatory, and anti-cell death pathways [71,72,73,74]. In 2018, using an ovariectomized (OVX) rat CIRI model, Rutin treatment was shown to upregulate the levels of activated estrogen receptor α (ERα), ERβ, brain-derived neurotrophic factor (BDNF), nerve growth factor (NGF), tropomyosin receptor kinase A (TrkA), TrkB, and phosphorylated cAMP response element-binding protein (p-CREB) in the hippocampus and cerebral cortex. The protective effects were partially abolished by the endoplasmic reticulum antagonist ICI182780, suggesting that Rutin pretreatment ameliorates CIRI in OVX rats via ER-mediated BDNF-TrkB and NGF-TrkA signaling [5]. Additionally, Rutin enhanced the viability of PC12 neurons under serum/glucose deprivation (SGD) conditions, reducing ROS production and lipid peroxidation. It also inhibited apoptosis by downregulating pro-apoptotic proteins (Bax, caspase-3, caspase-9) and upregulating the anti-apoptotic protein Bcl-2 [75]. Regarding pyroptosis, Troxerutin (TXN) was found to downregulate the expression of pyroptosis-related proteins (NLRP3, caspase-1, ASCI) and Toll-like receptor 3 (TLR3), while reducing inflammatory markers (IL-6, IL-4, TNF-α), thereby improving IRI outcomes [76]. Recent studies have focused on innovative delivery systems and refined molecular mechanisms. A 2023 study developed a Rutin-based three-target self-assembling nanodelivery system (SHR) for ischemic stroke. This system demonstrated high affinity for the ACE2 receptor, achieving vascular normalization and anti-inflammatory effects via the activation of ACE2/Ang1-7 signaling, which effectively reduced neurological deficits and infarct size in tMCAO rats [77]. In the same year, another study found that the co-administration of Rutin and Lithium exerted synergistic protective effects in a global CIRI rat model. This combination significantly attenuated the mRNA transcription of antioxidant markers (Hmox1 and Nqo1) and pro-inflammatory markers (IL-2, IL-6, and IL-1β). Mechanistically, this was achieved by inhibiting GSK-3β phosphorylation, upregulating downstream β-catenin and Nrf2 expression, suppressing NF-κ-induced inflammation, and enhancing the expression of key neuroprotective proteins such as CREB and BDNF [78]. In summary, Rutin exerts comprehensive neuroprotection in CIRI through multi-target mechanisms, holding significant promise for stroke therapy, especially within the realms of nanomedicine and combination regimens.

### 4.2. Protective Effects of Rutin in Myocardial Ischemia–Reperfusion Injury (MIRI)

The cardioprotective effects of the flavonoids Rutin and Troxerutin against MIRI have been extensively investigated, primarily utilizing rat models and H9c2 CMs for both in vivo and in vitro studies [79]. Early research confirmed that Rutin protects isolated rat hearts from MIRI via antioxidant mechanisms [80]. Specifically, Rutin was shown to significantly reduce the percentage of left ventricular necrosis (PLVN) and lipid peroxidation products, while bolstering myocardial antioxidant defenses, including catalase (CAT) and reduced GSH [81]. Mechanism-focused studies from the same period demonstrated that Rutin preserves myocardial contractile function and exerts anti-apoptotic effects by elevating the Bcl-2/Bax ratio and suppressing active Caspase-3. In an in vitro H9c2 cell injury model, these protective benefits were shown to be dependent on the ERK1/2 and PI3K/Akt signaling pathways, as evidenced by the blocking effects of specific inhibitors [82]. A 2011 study indicated that Rutin limited infarct size in both normal and diabetic rats. Notably, the cardioprotective effect of Rutin was partially abolished by the pre-administration of L-NAME, a NO synthase inhibitor, suggesting a partial role for NO in this mechanism [83]. Research in 2012 focused on hemodynamics, demonstrating that Rutin possesses potent free radical scavenging activity and protects hemodynamic function in isolated rat hearts via antioxidant activity. Rutin significantly reduced left ventricular end-diastolic pressure (LVEDP) and markedly improved the maximum rate of rise/fall of left ventricular pressure (±dP/dt), while increasing superoxide dismutase (SOD) activity in both H9c2 cells and primary CMs [84]. In 2015, continuous oral administration of Troxerutin was shown to prevent MIRI in isolated hearts from healthy and diabetic rats. Troxerutin significantly lowered levels of cardiac troponin I (cTnI), a marker of myocardial injury, and reduced tissue apoptosis. This mechanism was linked to the inhibition of GSK-3β activity via phosphorylation [85]. Concurrently, another study examining Rutin’s protection in isolated rat hearts found that it attenuated infarct size and reduced oxidative stress (indicated by decreased TBARS and increased GSH). Interestingly, this study showed that rutin significantly reduced IRI-induced Na^+^/K^+^-ATPase activity. The effect of rutin is likely similar to that of ouabain (a selective Na KT ATPase inhibitor), which acts as a buffer against calcium overload during ischemia through other signaling pathways or by initiating pretreatment protection [86]. Two studies in 2017 further established the mechanism of action for Troxerutin. Troxerutin pretreatment was confirmed to mediate cardioprotection via the PI3K/Akt pathway, as the inhibitor LY294002 blocked its protective effects [87]. Furthermore, both Troxerutin pretreatment and post-ischemic conditioning significantly reduced the post-IRI inflammatory response, including the suppression of pro-inflammatory cytokines (TNF-α and IL-1β) and ICAM-1 activity [88]. A 2018 study also confirmed Troxerutin’s significant anti-arrhythmic and anti-inflammatory effects (lowering TNF-α, IL-1β, and ICAM-1) in a diabetic rat model [89]. At the molecular level, research in 2019 revealed that Troxerutin attenuated apoptosis in rat hypoxia/reoxygenation (H/R) cardiomyocyte models and rat IRI models by inhibiting miR-146a-5p transcription [90]. Another 2019 study found that Rutin alleviated H/R injury and oxidative stress by upregulating SIRT1 expression, an effect blocked by SIRT1 inhibitors [91]. In 2020, the mechanism of Troxerutin was further refined, showing it mitigates oxidative stress and inflammation by enhancing the PI3K/AKT/HIF-1α signaling pathway [92]. The latest research reveals that Rutin protects the myocardium by targeting the NF-κB/NLRP3/pyroptosis pathway. Rutin reduced infarct size, oxidative stress, and the release of inflammatory factors (TNF-α, IL-1β, IL-18). It inhibited pyroptosis by downregulating the expression of NF-κB, NLRP3, active Caspase-1, and cleaved GSDMD protein. The abolition of Rutin’s protection by an NLRP3 agonist reaffirmed the central role of this pathway [1]. The protective effects of Rutin in MIRI are shown in Table 2.

### 4.3. Protective Effects of Rutin in Hepatic IRI (Transplantation and Surgical Models)

Rutin demonstrated multifaceted mechanisms and therapeutic implications in metabolic dysfunction-associated fatty liver disease (MAFLD) [93]. A 2007 study confirmed that Rutin significantly attenuates hepatic I/R injury in rats, with mechanisms involving antioxidant activity and the modulation of the DDAH/NOS pathway. Rutin treatment normalized elevated liver injury markers (ALT and AST), inhibited lipid hydroperoxide (LOOH) formation and DNA fragmentation, and replenished thiol group (RSH) levels [7]. In 2009, a subsequent study investigated the efficacy of the co-administration of Rutin and L-arginine. The results indicated that this combined treatment was more effective in mitigating liver injury—as evidenced by elevated ALT/AST activities—than either Rutin or L-arginine monotherapy. The protective effect of this combination was associated with the significant induction of heme oxygenase-1 (HO-1) expression and the inhibition of iNOS activity, suggesting that HO-1 induction may be one of the mechanisms underlying its beneficial effects [94]. Shifting focus to distant organ injury, a 2023 study examined oxidative damage in the lungs induced by hepatic IRI. The findings demonstrated that Rutin significantly prevented lung injury and exerted antioxidant and anti-inflammatory protection by reducing malondialdehyde (MDA) and MPO—an indicator of polymorphonuclear leukocyte (PMNL) activation—in both blood and lung tissues, while increasing total glutathione (tGSH) levels. The study concluded that Rutin holds potential for preventing distant organ (lung) injury caused by hepatic IRI [7]. The Protective effects of Rutin in hepatic IRI is shown in Table 3.

### 4.4. Protective Effects of Rutin in Renal IRI

Accumulating evidence substantiates the protective role of Rutin in a spectrum of renal injury models, encompassing renal IRI, obstructive nephropathy, and drug-induced or septic renal damage. The mechanisms underpinning this protection are multifaceted, involving the scavenging of ROS, inhibition of the NF-κB and TGF-β/Smad signaling axes, suppression of inflammation and apoptosis, and the enhancement of mitochondrial function [96,97,98]. Clinically, Rutin treatment consistently correlates with reduced serum creatinine and blood urea nitrogen (BUN) levels, alongside ameliorated histopathological changes. In the specific context of IRI, Rutin has demonstrated robust efficacy. Korkmaz et al. (2010) utilized a rat model (unilateral nephrectomy + 45 min ischemia/3 h reperfusion) to show that intraperitoneal Rutin (1 g/kg) significantly attenuated serum creatinine, BUN, and LDH, while restoring renal MnSOD activity and GSH levels, thereby mitigating oxidative damage [99]. Building on this, their 2013 study identified the inhibition of nitrosative stress as a key mechanism, observing that Rutin suppressed iNOS activity, 3-nitrotyrosine (3-NT) formation, and plasma NO metabolite/cGMP levels [100]. In 2015, Muthuraman et al. established an isolated rat kidney IRI model using a Langendorff perfusion system. Following oral administration of Rutin (100 mg/kg) for five days, results showed significantly reduced levels of creatinine, urea, and creatine kinase (CK) in the perfusate. The study indicated that Rutin inhibited Na^+^-K^+^-ATPase activity, reduced TBARS generation, and restored GSH levels, suggesting protection through antioxidant activity and the modulation of ion pump activity [101]. In 2022, Güzel et al. employed a rat model of 30 min renal ischemia and 1 h reperfusion with Rutin pretreatment (500 mg/kg, i.g.) [6]. Rutin significantly decreased serum BUN, urea, and TNF-α levels, reduced total oxidant status (TOS) and the apoptosis index in renal tissue, and improved histopathological outcomes, thereby demonstrating protection via anti-inflammatory, antioxidant, and anti-apoptotic mechanisms. Most recently, in 2024, Feng et al. developed Rutin-loaded polydopamine nanoparticles (PPR NPs). Intravenous injection in a mouse IRI model showed that PPR NPs could target the kidney, scavenge ROS, repair mitochondrial function, and inhibit ferroptosis, significantly improving renal function and histological damage. This highlights the innovative therapeutic potential of Rutin nanoformulations in IRI [102]. Collectively, these findings underscore Rutin’s potent renoprotective potential in IRI through a complex network of antioxidant, anti-inflammatory, and anti-ferroptotic mechanisms, paving the way for future clinical translation. The protective effects of Rutin in renal IRI are shown in Table 4.

### 4.5. Protective Effects of Rutin in IRI of Other Organs

Rutin exerts comprehensive protective effects against IRI across multiple organ systems—including the testes, ovaries, gastric mucosa, skeletal muscle, and retina—mediated by its antioxidant, anti-inflammatory, and anti-apoptotic activities. Highlighting its therapeutic potential in male infertility, studies in rat testicular IRI models demonstrate that Rutin significantly alleviates oxidative stress and tissue damage [103,104]. This is achieved by scavenging ROS, reducing MDA levels, and boosting the activity of endogenous antioxidant enzymes (SOD and CAT), leading to improved long-term spermatogenic function [103,105]. Similarly, Rutin exerts beneficial effects on the female reproductive system [106]. In ovarian IRI models, Rutin significantly downregulated MDA levels, TNF-α and IL-1β levels, and COX-2 activity, while elevating tGSH levels and COX-1 activity in ovarian [107].

In the context of the digestive system, Rutin provides dose-dependent protection against gastric mucosal injury [108]. Its mechanism involves antioxidant activity (reduction in MDA), the inhibition of neutrophil infiltration (reduced MPO activity), and the modulation of the NOS/NO pathway—specifically, the maintenance of constitutive NOS (cNOS) activity and the inhibition of inducible NOS (iNOS) [109].

Rutin exerts multifaceted protective effects on skeletal muscle by regulating oxidative stress, inflammation, and mitochondrial biogenesis via the activation of the AMPK/PGC-1α pathway. These molecular actions translate into improved muscle architecture, augmented exercise capacity, and reduced fatigue in both aging and injured states [108,110,111,112]. In the specific context of skeletal muscle IRI, Rutin treatment significantly lowers serum injury markers (CPK and LDH). This protection is mediated by the suppression of oxidative stress (enhancing total antioxidant status) and inflammation (reducing TNF-α, IL-1β, IL-6), as well as the inhibition of leukocyte recruitment via the downregulation of adhesion molecules (E-selectin, L-selectin, and ICAM-1) [2]. Furthermore, the enzyme-flavonoid complex Phlogenzym^®^ (containing Rutin, trypsin, and bromelain) has been shown to improve microvascular hemodynamics and prevent lipid peroxidation (plasma MDA elevation) in a rabbit model of IRI [113].

Regarding ocular neuroprotection, Rutin dose-dependently preserves the viability of retinal ganglion cells (RGCs) in in vitro H/R models. Mechanistically, Rutin drives microglial polarization toward the anti-inflammatory M2 phenotype and impedes the JAK/STAT3 signaling axis by selectively inhibiting JAK1 phosphorylation. This modulation effectively suppresses the secretion of key pro-inflammatory cytokines, including TNF-α and IL-6 [114]. The protective effects of Rutin in IRI of other organs are shown in Table 5.

## 5. Molecular Mechanisms Underlying Rutin-Mediated Protection Against IRI

Rutin and its derivative, Troxerutin, have demonstrated broad-spectrum protective effects across multiple organ IRI models, encompassing vital organs such as the brain, heart, kidney, and liver. These protective effects stem from the multidimensional regulation of complex molecular networks, including the inhibition of oxidative stress, the attenuation of inflammatory responses, the blockade of apoptosis and cell death, and the modulation of various critical signaling pathways. Furthermore, recent studies have revealed that Rutin can specifically target mitochondrial dysfunction and novel cell death pathways—such as pyroptosis and ferroptosis—providing a more precise theoretical basis for its clinical application.

### 5.1. Modulation of Oxidative Stress

The core mechanism underlying Rutin’s protective efficacy against IRI pivots on its robust antioxidant capability. Specifically, this action encompasses the direct scavenging of ROS, the upregulation of endogenous antioxidant defenses, and the modulation of nitrosative stress.

#### 5.1.1. Direct Scavenging and Inhibition of ROS Generation

Rutin has been confirmed to possess potent free radical scavenging activity. Across IRI models involving the brain, myocardium, kidney, liver, testes, ovaries, stomach, and skeletal muscle, Rutin significantly lowers the levels of lipid peroxidation products, such as MDA and thiobarbituric acid reactive substances (TBARS) [2]. Furthermore, in the context of CIRI, Rutin has been shown to reduce H2O2 and PC levels [66]. Of particular note in renal IRI, Rutin-loaded polydopamine nanoparticles (PPR NPs) have shown the capacity to specifically target renal tissue and scavenge mitochondrial ROS (mitoROS), consequently lowering the TOS [102].

#### 5.1.2. Activation of Endogenous Antioxidant Systems

Rutin possesses the capacity to restore and enhance the activity of endogenous antioxidant enzymes. In the context of cerebral IRI, Rutin significantly elevates the activities of glutathione peroxidase (GPx), glutathione reductase (GR), CAT, and SOD [1,67,103]. In myocardial tissue, Rutin increases SOD activity and improves the status of CAT and reduced GSH [86]. In renal IRI, Rutin facilitates the restoration of manganese superoxide dismutase (MnSOD) activity and GSH levels [99]. Regarding hepatic IRI, rutin exerts protective effects by replenishing thiol group (RSH) levels and increasing tGSH concentrations [95]. Furthermore, in testicular and ovarian IRI models, Rutin significantly boosts SOD and CAT activities while elevating tGSH levels [105,109].

#### 5.1.3. Modulation of Nitrosative Stress

Rutin mitigates nitrosative stress through the differential modulation of NOS isoforms. In the context of CIRI, Rutin orchestrates NOS activity by downregulating neuronal NOS (nNOS) and iNOS, while conversely upregulating endothelial NOS (eNOS) [74]. This protective regulatory pattern is also observed in renal IRI, where Rutin significantly suppresses iNOS activity and limits 3-NT formation, thereby conferring renoprotection [100]. In hepatic IRI, Rutin modulates the DDAH/NOS axis to inhibit iNOS and induce eNOS [7]. Furthermore, in gastric mucosal IRI, Rutin acts to preserve the activity of cNOS while concurrently inhibiting iNOS activity [109].

### 5.2. Inhibition of Inflammatory Responses

Rutin attenuates IRI-induced tissue damage by suppressing inflammatory responses via multiple pathways—a therapeutic effect validated across diverse organ systems including the heart, brain, kidney, ovary, skeletal muscle, and retina.

#### 5.2.1. Regulation of Inflammatory Mediators and Cytokines

Generally, Rutin treatment results in a marked reduction in post-IRI inflammation. Specifically, in the context of CIRI, Rutin decreases serum concentrations of the pro-inflammatory cytokines TNF-α and IL-1β, reduces MPO levels, and lowers IL-6 [71]. Similarly, in MIRI, Rutin or Troxerutin inhibits the activity of TNF-α and IL-1β while downregulating IL-6 and IL-18 [1]. This anti-inflammatory profile extends to renal and ovarian IRI, where Rutin significantly lowers TNF-α and IL-1β levels [107]. In skeletal muscle IRI, Rutin reduces the release of inflammatory factors such as TNF-α and IL-1β, and IL-6 [2]. Finally, in retinal IRI, Rutin suppresses the release of pro-inflammatory factors including TNF-α and IL-6 [114].

#### 5.2.2. Inhibition of Inflammatory Cell Recruitment and Adhesion Molecules

Rutin confers tissue protection primarily by suppressing the recruitment and activation of inflammatory cells. In the context of cerebral IRI, it lowers pathological levels of MMP-9, downregulates adhesion molecules (ICAM-1 and ICAM-2), and exerts an inhibitory effect on microglial activation [69]. Regarding hepatic IRI, Rutin acts to prevent secondary distant organ injury—specifically in the lungs—by reducing MPO levels. A similar mechanism is observed in gastric mucosal IRI, where Rutin impedes neutrophil infiltration as evidenced by decreased MPO activity [95]. In skeletal muscle IRI, Rutin effectively blocks leukocyte adhesion and infiltration through the downregulation of E-selectin, L-selectin, and ICAM-1 [2]. Furthermore, in retinal IRI, Rutin facilitates the polarization of microglia toward the anti-inflammatory M2 phenotype, thereby actively resolving inflammation [114].

### 5.3. Attenuation of Apoptosis and Cell Death

Rutin confers robust cytoprotection by regulating apoptosis-associated genes, blocking the mitochondrial intrinsic apoptotic pathway, and suppressing emerging cell death modalities, including pyroptosis and ferroptosis.

#### 5.3.1. Regulation of Canonical Apoptotic Pathways

It exerts a significant anti-apoptotic effect across diverse organ systems. Mechanistically, in both myocardial and cerebral IRI models, Rutin shifts the apoptotic balance toward survival by upregulating the anti-apoptotic protein Bcl-2 and downregulating the pro-apoptotic protein Bax, thereby increasing the Bcl-2/Bax ratio [75,82]. Concurrently, it reduces the cleavage of active Caspase-3 and Caspase-9. In the specific context of cerebral IRI, Rutin further alleviates DNA damage and arrests apoptosis via the downregulation of p53 expression [75,82].

#### 5.3.2. Inhibition of Pyroptosis and Ferroptosis

Recent mechanistic investigations into Rutin have pivoted toward its role in suppressing inflammasome-mediated cell death. In models of myocardial and cerebral IRI, Rutin exerts protective effects by targeting the NF-κB/NLRP3/pyroptosis axis [1,76]. Mechanistically, Rutin mitigates pyroptosis by downregulating the expression of NLRP3, active Caspase-1, and cleaved GSDMD. In parallel, Troxerutin improves outcomes in CIRI by downregulating ASC and TLR3, thereby exerting anti-pyroptotic activity [76]. Regarding renal IRI, Rutin-loaded nanoparticles (PPR NPs) have been shown to suppress ferroptosis, a novel cell death modality, via the upregulation of GPX4 [102].

### 5.4. Modulation of Key Signaling Pathways

At the molecular level, Rutin mediates protective effects by activating cell survival signaling pathways, suppressing inflammatory cascades, and regulating hormone- and metabolism-related pathways.

#### 5.4.1. Activation of Cell Survival Signaling Pathways

In the context of MIRI, the protective effects of Rutin are achieved through the activation of the ERK1/2 and PI3K/Akt signaling pathways [82]. Similarly, Troxerutin mediates cardioprotection via the PI3K/Akt axis and enhances the PI3K/Akt/HIF-1α signaling pathway to mitigate oxidative stress and inflammation [92]. In both myocardial and cerebral tissues, Troxerutin functions by phosphorylating GSK-3β, thereby inhibiting its activity [78,85]. In the brain, the co-administration of Rutin and Lithium upregulates downstream Nrf2 protein expression by inhibiting GSK-3β phosphorylation [78]. In skeletal muscle, Rutin activates the AMPK/PGC-1α pathway, thereby enhancing mitochondrial biogenesis [110].

#### 5.4.2. Inflammatory and Protective Transcription Factor Pathways

Rutin exerts significant regulatory control over the NF-κB pathway. It downregulates NF-κB expression in the myocardium and kidney [96], while in the brain, it potentially attenuates inflammation by inhibiting the RAGE-NF-κB signaling axis [70]. Regarding antioxidant signaling, the synergistic co-administration of Rutin and Lithium in cerebral IRI potentiates downstream Nrf2 protein expression [78]; similarly, Rutin induces HO-1 expression in hepatic tissues [94]. Furthermore, in retinal IRI, Rutin impedes the JAK/STAT3 signaling cascade via the selective inhibition of JAK1 phosphorylation, effectively suppressing the release of pro-inflammatory cytokines [114].

#### 5.4.3. Estrogen Receptor-Mediated Neuroprotection

In the context of CIRI in OVX rats, Rutin has been shown to elevate the levels of activated estrogen receptors, specifically ERα and ERβ [5]. Acting through ER-mediated signaling cascades, Rutin stimulates the BDNF-TrkB and NGF-TrkA axes, leading to the upregulation of key neuroprotective proteins such as CREB and BDNF.

**Figure 3:**
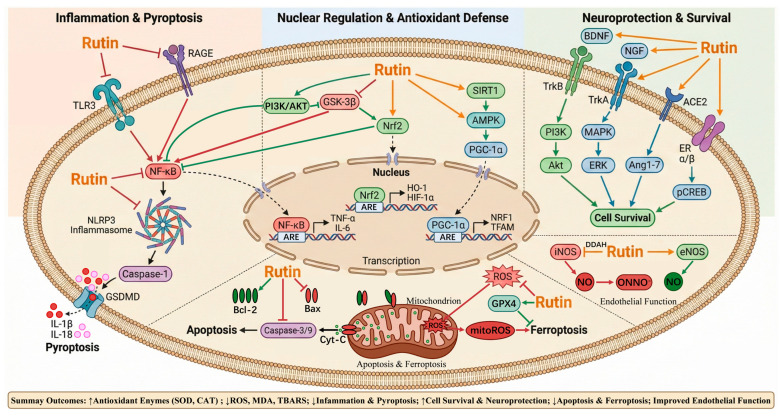
The main mechanism of rutin in preventing ischemia–reperfusion injury.

In summary, Rutin exerts protection against IRI through a mechanism that is as complex as it is comprehensive. Acting as a versatile ‘molecular toolkit,’ it orchestrates a multifaceted defense by simultaneously triggering the cell’s ‘firefighting’ capacity (antioxidant activity) and ‘emergency response’ systems (anti-apoptotic and anti-inflammatory regulation). This dual approach effectively mitigates the ‘double hit’ of ischemia–reperfusion, ensuring the maximal preservation of cellular and organ architecture and function. The main mechanism of rutin in preventing IRI is shown in Figure 3.

## 6. Challenges and Future Perspectives

Rutin, a potent natural flavonoid, demonstrates substantial clinical prospects and therapeutic efficacy in protecting a broad spectrum of organs—including the kidney, heart, brain, liver, testes, ovaries, and skeletal muscle—from IRI. The field is currently witnessing a paradigm shift toward advanced delivery systems and the refinement of molecular targets. In the context of renal IRI, the development of Rutin-loaded polydopamine nanoparticles (PPR NPs) represents a significant breakthrough. These nanoparticles effectively target renal tissue, scavenge ROS, repair mitochondrial function, and suppress ferroptosis, underscoring the potential of Rutin nano-formulations [102]. Similarly, for CIRI, a three-target self-assembling nanodelivery system (SHR) has been engineered to activate the ACE2/Ang1-7 axis, facilitating vascular normalization and anti-inflammatory responses [77]. Such cutting-edge delivery platforms are poised to surmount the limitations of bioavailability and targeting specificity inherent in conventional administration. In parallel, combination therapies have emerged as a promising strategy for synergistic protection. Notably, the co-administration of Rutin and Lithium has been shown to markedly reduce inflammatory markers in CIRI via GSK-3β inhibition and Nrf2 upregulation [78].

Mechanistically, Rutin’s profile is becoming increasingly well-defined: it targets the NF-κB/NLRP3/pyroptosis pathway in MIRI [1]; activates ER-driven neurotrophic signaling (BDNF-TrkB/NGF-TrkA) in CIRI [5]; and mitigates remote organ injury (e.g., pulmonary oxidative stress) following hepatic IRI [95]; additionally, its protective role in testicular IRI suggests a noteworthy therapeutic avenue for male infertility [104].

However, several challenges must be addressed to bridge the translational gap. Most findings are currently derived from preclinical models. Realizing the full clinical potential of Rutin necessitates rigorous human clinical trials to validate its safety and efficacy, alongside the standardization of indications, dosing regimens, and routes of administration. Since current studies utilize diverse protocols (e.g., oral, i.p., i.v.), future efforts must prioritize the optimization of pharmacokinetics and targeting efficiency to facilitate successful clinical translation.

## 7. Conclusions

Rutin demonstrates comprehensive and robust protection against IRI across a spectrum of vital organs, including the kidney, heart, brain, liver, testes, and skeletal muscle. The core therapeutic superiority of Rutin is attributed to its pleiotropic, multi-targeted mechanism. Mechanistically, this involves the orchestrated mitigation of oxidative stress (characterized by ROS scavenging, elevated SOD/GSH, and decreased MDA/TBARS), the suppression of inflammation (via NF-κB inhibition and reduced TNF-α/IL-1β), and the blockade of regulated cell death modalities (apoptosis, pyroptosis, and ferroptosis). Through these concerted actions, Rutin effectively attenuates injury biomarkers (such as creatinine, BUN, CK, and ALT/AST), improves histopathological outcomes, and promotes functional restoration. Thus, Rutin and its derivatives represent a class of high-potential neuroprotective and organ-protective agents for ischemic diseases, especially when leveraged through advanced delivery systems or combination therapies. Metaphorically acting as a multifunctional ‘rescue team,’ Rutin extinguishes the ‘fire’ of oxidative stress, dissipates the ‘smoke’ of inflammation, and repairs cellular damage, thereby ensuring that organs can maximally recover physiological function following the dual insult of ischemia and reperfusion.

## Figures and Tables

**Table 1:** Protective effects of Rutin in CIRI.

Experimental Model	Dose and Administration	Effects	Key Pathways/Mechanisms	Ref.
Rat CIRI	50 mg/kg (ip)	Ameliorated spatial memory impairment; Neuronal death in hippocampal CA1 region↓	Anti-apoptotic	[64]
Rat CIRI	25 mg/kg (ip), 21-day pretreatment	GPx↑, GR↑, CAT↑, SOD↑, GSH↑; TBARS↓, H_2_O_2_↓, PC↓; p53↓; Apoptosis↓	Antioxidant	[66]
Rat CIRI	100 mg/kg (ip)	GPx↑,GR↑, SOD↑, MDA↓; TNF-α↓, IL-1β↓; LDH↓, Ca^2+^↓; Ameliorated brain injury	Antioxidant, Anti-inflammatory	[67]
Rat CIRI	10 mg/kg (ip) 10 min pretreatment	SOD↑, CAT↑, MDA↓; MPO↓; Infarct size↓	Antioxidant, Anti-inflammatory	[68]
Rat CIRI	50 mg/kg (ip)	MMP-9↓; BBB permeability↓	Improved functional outcomes	[69]
Rat SAH	50 mg/kg (ip)	RAGE↓, NF-κB↓; BBB permeability↓	Anti-inflammatory	[70]
NVU with OGD/R injury	10–1000 µM	GAP-43↑, Claudin-5↑, AQP-4↓; TNF-α↓, IL-1β↓, IL-6, ↓VCAM-1↓; Bax↓, p53↓, caspase-1↓; Maintained normal BBB structure	Anti-inflammatory, Anti-cell death	[71]
Rat CIRI	2.0 mL/kg (ip)	LD↑, LDH↓; SOD↑, MDA↓; Promoted endothelial cell proliferation, adhesion, migration, and angiogenesis	Antioxidant, Pro-angiogenic	[72]
Rat CIRI	2.0 mL/kg (ip)	caspase-1↓, aspase-3↓, caspase-8↓	Anti-cell death	[73]
Rat CIRI	2.0 mL/kg (ip) 5-day pretreatment	nNOS↓, iNOS↓, eNOS↑; Modulated activity of NOS isoforms	Antioxidant	[74]
OVX Rat IRI	100 mg/kg (ip) 5-day pretreatment	ERα↑, ERβ↑, BDNF↑, NGF↑, TrkA↑, TrkB↑, pi-CREB↑; Attenuated neuronal loss	Activation of ER-mediated signaling	[5]
PC12 cells (SGD)	0–200 µM	ROS↓, Lipid peroxidation↓; Bax↓, Bcl-2↑, caspase-3↓, caspase-9↓	Antioxidant, Anti-apoptotic	[75]
Rat CIRI	20 mg/kg (ip) 15 min pretreatment	TBARS↓, LOOH↓, SOD↑, MDA↓, GSH↑; IL-6↓, IL-4↓, TNF-α↓; NLRP3↓, caspase-1↓, ASCI↓, TLR3↓;	Antioxidant, Anti-inflammatory, Anti-pyroptotic	[76]
Rat CIRI	25 mg/kg (ip)	Ho-1↑, PSD-95↓; Hmox1↓, Nqo1↓; IL-2↓, IL-6↓, IL-1β↓piGsk-3β↓; β-catenin↑; Nrf2↑; pi-NF-kB↓; pi-CREB↑, BDNF↑; Ameliorated post-stroke neuroinflammation	Antioxidant, Anti-inflammatory, Neurotrophic	[78]

↑ indicates an increase, and ↓ indicates a decrease. The same applies to the following tables.

**Table 2:** Protective effects of Rutin in MIRI.

Experimental Model	Dose and Administration	Effects	Key Pathways/Mechanisms	Ref.
Isolated Rat MIRI	5 μM	Free radicals↓	Antioxidant	[80]
Rat MIRI	10 mg/kg (ip), 10 min pretreatment	MDA↓, CAT↓, GSH↑, SOD↑; AST/ALT↓; Infarct size↓	Antioxidant	[81]
Rat MIRI	10 mg/kg (ip), 1 h pretreatment	Bcl-2/Bax↑, Caspase-3↓; Myocardial contractile function↑, Infarct size↓	Anti-apoptotic	[82]
H9c2(H_2_O_2_ injury)	20 μM	Bcl-2/Bax↑, Caspase-3↓; pi-ERK↑; pi-Akt↑	Anti-apoptotic	[82]
Diabetic Rat MIRI	10 mg/kg (ip), 10 min pretreatment	NO↓; Infarct size↓	Vasodilation	[83]
Isolated Rat MIRI	50 µM	VEDP↓, ±dP/dt↑; SOD↑, DPPH↑; Cardiac dynamics↑	Antioxidant	[84]
Isolated MIRI (Healthy and Diabetic Rats)	150 mg/kg (po) 4-week pretreatment	cTnI↓; GSK-3β activity↓; Apoptosis index↓	Anti-apoptotic	[85]
Isolated Rat MIRI	150 mg/kg (po)	CK-MB↓, LDH1↓; TBARS↓, GSH↑; Na^+^-K^+^-ATPase↓; Infarct size↓; Improved coronary flow	Antioxidant	[86]
Rat MIRI	150 mg/kg (po)	CK↓, AST, LDH↓; TNF-α↓, IL-1β↓, IL-10↓; pi-PI3K↑pi-Akt↑; Bax↓,Caspase 3↓	Anti-inflammatory, Anti-apoptotic	[87]
Isolated Rat MIRI	150 mg/kg (po), 4-week pretreatment	CK↓; TNF-α↓, IL-1β↓, ICAM-1↓	Anti-inflammatory	[88]
Isolated MIRI (Diabetic Rats)	150 mg/kg (po), 4-week pretreatment	TNF-α↓, IL-1β↓, ICAM-1↓; Anti-arrhythmic effects	Anti-inflammatory	[89]
Rat MIRI; CMs (H/R)	150 mg/kg (po), 4-week pretreatment; 1–20 μM	CK↓, LDH; TNF-α↓, IL-10↓; Bcl-2/Bax↑, Caspase 3↓; MiR-146a-5p↓	Anti-inflammatory, Anti-apoptotic	[90]
H9c2(H/R)	50 μM	SOD↑, GSH-Px↑, MDA↓, GSH↑; Caspase 3↓,SIRT1↑; Apoptosis rate↓	Antioxidant, Anti-apoptotic	[91]
H9c2(H/R)	10 μM	SOD↑, GSH-Px↑, MDA↓; IL-1β↓, IL-6↓, TNF-α↓; PI3K↑, HIF-1α↑; pi-AKT/AKT ratio↑	Antioxidant, Anti-inflammatory, Anti-apoptotic	[92].
Mouse MIRI; CMs (H/R)		CK-MB↓, cTnT/I↓, MDA↓, ROS↓; IL-1β↓, TNF-α↓, IL-18↓; NF-κB↓, NLRP3↓, Caspase-1↓, GSDMD↓; Infarct size↓ Pyroptosis rate↓	Antioxidant, Anti-inflammatory, Anti-pyroptotic	[1]

**Table 3:** Protective effects of Rutin in hepatic IRI.

Experimental Model	Dose and Administration	Effects	Key Pathways/Mechanisms	Ref.
Rat Hepatic IRI	30 mg/kg (ip), 3-day pretreatment	ALT↓, AST↓, LOOH↓; DNA fragmentation ↓; RSH↑; iNOS↓, eNOS↑; DDAH-1↓	Antioxidant	[7]
Rat Hepatic IRI	30 mg/kg (ip), 3-day pretreatment	ALT↓, AST↓, LOOH↓; DNA fragmentation ↓; RSH↑; iNOS↓, HO-1↑	Antioxidant	[94]
Rat Hepatic IRI	50 mg/kg (ip), 1 h pretreatment	MDA↓; MPO↓; tGSH↑	Antioxidant, Anti-inflammatory	[95]

**Table 4:** Protective effects of Rutin in renal IRI.

Experimental Model	Dose and Administration	Effects	Key Pathways/Mechanisms	Ref.
Rat Renal IRI	1 g/kg (ip)	Creatinine↓, BUN↓; LDH↓, MDA; MnSOD↑, GSH↑, Tissue injury↓	Antioxidant	[99]
Rat Renal IRI	1 g/kg (ip)	iNOS↓, 3-NT↓, NO↓	iNOS/NO pathway modulation	[100]
Isolated Renal IRI	100 mg/kg (ip), 5-day pretreatment	Creatinine↓, BUN ↓, CK; Na^+^-K^+^-ATPase↓, TBARS↓, GSH↑	Antioxidant	[101]
Rat Renal IRI	500 mg/kg (ip)	BUN↓, BUN ↓; TNF-α↓, TOS↓; Apoptosis ↓; Improved histopathology	Antioxidant, Anti-inflammatory, Anti-apoptotic	[6]
Mouse Renal IRI	Rutin Nanoparticles (PPR NPs) (iv)	mitoROS↓, Mitochondrial function ↑; GPX4↑; Improved histopathology	Antioxidant, Anti-mitochondrial damage, Anti-ferroptotic	[102]

**Table 5:** Protective effects of Rutin in IRI of other organs.

Experimental Model	Dose and Administration	Effects	Key Pathways/Mechanisms	Ref.
Rat Testicular IRI	10 mg/kg (ip), 30 min pretreatment	MDA↓; SOD↑, CAT↑; Testicular tissue structure ↑, Spermatogenic cell alignment ↑	Antioxidant	[103]
Rat Testicular IRI	30 mg/kg (ip), 3-month pretreatment	MDA↓; SOD↑, CAT↑, Spermatogenic function ↑, Spermatogenesis ↑	Antioxidant	[105]
Rat Ovarian IRI	50 mg/kg (ip), 1 h pretreatment	MDA↓, SOD↑, COX-1↑, tGSH↑; TNF-α↓, IL-1β↓	Antioxidant, Anti-inflammatory	[107]
Rat Gastric IRI	50–200 mg/kg (po)	MDA↓; MPO↓, cNOS↑, iNOS↓; Gastric mucosal injury index↓	Antioxidant, Anti-inflammatory, NOS/NO system	[109]
Rat Hindlimb Muscle IRI	10–100 mg/kg (ip), 30 min pretreatment	CPK↓, LDH↓; TAS↑; TNF-α↓, IL-1β↓, IL-6↓; ICAM-1↓, E-selectin↓, L-selectin↓, Leukocyte adhesion and infiltration↓	Antioxidant, Anti-inflammatory	[2]
Rabbit Hindlimb Muscle IRI	Rutin complex (Phlogenzym) (ip), 30 min pretreatment	MDA↓, Blood flow and microvascular function↑	Antioxidant	[113]
RGCs (H/R)	1–5 μmol/L, 2 h pretreatment	TNF-α↓, IL-6↓; pi-JAK1↓; Apoptosis↓	Anti-inflammatory, Anti-apoptotic	[114]

## Data Availability

No new data were created or analyzed in this study. Data sharing is not applicable to this article.

## Abbreviations

The following abbreviations are used in this manuscript:

BH4	Tetrahydrobiopterin
BUN	Blood urea nitrogen
BBB	Blood–brain barrier
BDNF	Brain-derived neurotrophic factor
CTNI	Cardiac troponin I
CL	Cardiolipin
CMs	Cardiomyocytes
CAT	Catalase
CIRI	Cerebral ischemia–reperfusion injury
CircRNAs	Circular RNAs
CoQ	Coenzyme Q
ceRNA	Competitive endogenous RNA
cNOS	Constitutive NOS
CK	Creatine kinase
Cyt C	Cytochrome c
DAMPs	Damage-associated molecular patterns
DRP1	Dynamin-related protein 1
ETC	Electron transport chain
eNOS	Endothelial NOS
ERα	Estrogen receptor α
Fe^3+^	Ferric iron
Fe^2+^	Ferrous iron
FBs	Fibroblasts
GSDMD	Gasdermin D
GSH	Glutathione
GPX	Glutathione peroxidase
GPX4	Glutathione peroxidase 4
GR	Glutathione reductase
H_2_O_2_	Hydrogen peroxide
HO-1	Heme oxygenase-1
HK2	Hexokinase 2
H/R	Hypoxia/reoxygenation
iNOS	Inducible NOS
I/R	Ischemia–reperfusion
IRI	Ischemia–reperfusion injury
LDH	Lactate dehydrogenase
LVEDP	Left ventricular end-diastolic pressure
LOOH	Lipid hydroperoxide
LOXS	Lipoxygenases
LncRNAs	Long non-coding RNAs
MDA	Malondialdehyde
MNSO	Manganese superoxide dismutase
MMP-9	Matrix metalloproteinase-9
MiRNAs	MicroRNAs
MVO	Microvascular obstruction
Mt-DNA	Mitochondrial DNA
MPTP	Mitochondrial permeability transition pore
MPO	Myeloperoxidase
MIRI	Myocardial IRI
NOX	NADPH oxidase
NGF	Nerve growth factor
nNOS	Neuronal NOS
NETs	Neutrophil extracellular traps
NO	Nitric oxide
NOS	Nitric oxide synthase
NcRNAs	Non-coding RNAs
O^2−^	Superoxide anions
OH^−^	Hydroxyl radicals
OVX	Ovariectomized
PLVN	Percentage of left ventricular necrosis
p-CREB	Phosphorylated camp response element-binding protein
PMNL	Polymorphonuclear leukocyte
PCs	Protein carbonyl
RNS	Reactive nitrogen species
ROS	Reactive oxygen species
RISK	Reperfusion injury salvage kinase
RSH	Replenished thiol group
RGCs	Retinal ganglion cells
SIgA	Secretory IgA
SA3K	Serpina3k
SGD	Serum/glucose deprivation
SCFAs	Short-chain fatty acids
SAH	Subarachnoid hemorrhage
SDH	Succinate dehydrogenase
SOD	Superoxide dismutase
SAFE	Survivor activating factor enhancement
TBARs	Thiobarbituric acid reactive substances
TLR3	Toll-like receptor 3
TGSH	Total glutathione
TOS	Total oxidant status
TCA	Tricarboxylic acid
TRKA	Tropomyosin receptor kinase A
TXN	Troxerutin
TCHI	Troxerutin-cerebroprotein hydrolysate injections
XO	Xanthine oxidase
γ-GT	γ-glutamyl transferase
3-NT	3-nitrotyrosine

## References

[1] Tian K., Song L., Liu L., Lai T., Liu W. (2025). Rutin Protects Myocardial Ischemia-Reperfusion Injury via the NF-κB/NLRP3/Pyroptosis Pathway. ACS Omega.

[2] Ergün Y., Baykişi Y., Kılınç M., Toksözlü F., Aykan D.A., Mavigök A.N., Eser N. (2026). Rutin and skeletal muscle ischemia-reperfusion injury: Effects of acute treatment. Tissue Cell.

[3] Sun Z., Wang X., Pang X. (2025). Potential of Polydatin Against Ischemia-Reperfusion Injury: New Insights from Pharmacological-Pathological Mechanism Associations. Drug Des. Devel Ther..

[4] Goyal J., Verma P.K. (2023). An Overview of Biosynthetic Pathway and Therapeutic Potential of Rutin. Mini Rev. Med. Chem..

[5] Liu H., Zhong L., Zhang Y., Liu X., Li J. (2018). Rutin attenuates cerebral ischemia-reperfusion injury in ovariectomized rats via estrogen-receptor-mediated BDNF-TrkB and NGF-TrkA signaling. Biochem. Cell Biol..

[6] Güzel A., Özorak A., Oksay T., Öztürk S.A., Bozkurt K.K., Yunusoğlu S., Uz E., Uğuz A.C., Aslan Koşar P. (2022). The efficiency of oxerutin on apoptosis and kidney function in rats with renal ischemia reperfusion injury. Ulus. Travma Acil Cerrahi Derg..

[7] Lanteri R., Acquaviva R., Di Giacomo C., Sorrenti V., Li Destri G., Santangelo M., Vanella L., Di Cataldo A. (2007). Rutin in rat liver ischemia/reperfusion injury: Effect on DDAH/NOS pathway. Microsurgery.

[8] Zhou T., Wang H., Zhang J., Bao Y., Yu Y., Chang J. (2025). Research progress in natural sources, biosynthesis and metabolism regulation of rutin in plant-derived food materials. Food Sci..

[9] Calabrese E.J., Pressman P., Hayes A.W., Dhawan G., Kapoor R., Agathokleous E., Calabrese V. (2024). RUTIN, a widely consumed flavonoid, that commonly induces hormetic effects. Food Chem. Toxicol..

[10] Chunmei Z., Shuai W. (2025). Molecular mechanisms of neuroprotective effect of rutin. Front. Pharmacol..

[11] Forouzanfar F., Pourbagher-Shahri A.M., Ahmadzadeh A.M. (2025). Rutin attenuates complete Freund’s adjuvant-induced inflammatory pain in rats. Iran. J. Basic Med. Sci..

[12] Abdullah H.A., Moawed F.S., Ahmed E.S., Abdel Hamid F.F., Haroun R.A. (2025). Iron chelating, antioxidant and anti-apoptotic activities of hesperidin and/or rutin against induced-ferroptosis in heart tissue of rats. Int. J. Immunopathol. Pharmacol..

[13] Liu H., Xu Q., Wufuer H., Li Z., Sun R., Jiang Z., Dou X., Fu Q., Campisi J., Sun Y. (2024). Rutin is a potent senomorphic agent to target senescent cells and can improve chemotherapeutic efficacy. Aging Cell.

[14] Wang M., Ma X., Gao C., Luo Y., Fei X., Zheng Q., Ma X., Kuai L., Li B., Wang R. (2023). Rutin attenuates inflammation by downregulating AGE-RAGE signaling pathway in psoriasis: Network pharmacology analysis and experimental evidence. Int. Immunopharmacol..

[15] Chen S., Tang Y., Gao Y., Nie K., Wang H., Su H., Wang Z., Lu F., Huang W., Dong H. (2022). Antidepressant Potential of Quercetin and its Glycoside Derivatives: A Comprehensive Review and Update. Front. Pharmacol..

[16] Foudah A.I., Alqarni M.H., Alam A., Devi S., Salkini M.A., Alam P. (2022). Rutin Improves Anxiety and Reserpine-Induced Depression in Rats. Molecules.

[17] Liu Y., Sun Z., Dong R., Liu P., Zhang X., Li Y., Lai X., Cheong H.F., Wu Y., Wang Y. (2024). Rutin ameliorated lipid metabolism dysfunction of diabetic NAFLD via AMPK/SREBP1 pathway. Phytomedicine.

[18] Chekuri S., Sirigiripeta S.R., Thupakula S., Vyshnava S.S., Ayesha S., Karamthote J., Cheniya S.B., Kuruva R., Anupalli R.R. (2025). Rutin isolated from Acalypha indica L.: A comprehensive analysis of its antibacterial and anticancer activities. Biochem. Biophys. Res. Commun..

[19] Forouzanfar F., Sahranavard T., Tsatsakis A., Iranshahi M., Rezaee R. (2025). Rutin: A pain-relieving flavonoid. Inflammopharmacology.

[20] Ma X., Ren X., Zhang X., Wang G., Liu H., Wang L. (2024). Rutin ameliorate PFOA induced renal damage by reducing oxidative stress and improving lipid metabolism. J. Nutr. Biochem..

[21] Kessas K., Lounis W., Chouari Z., Vejux A., Lizard G., Kharoubi O. (2024). Benefits of rutin on mitochondrial function and inflammation in an aluminum-induced neurotoxicity rat model: Potential interest for the prevention of neurodegeneration. Biochimie.

[22] Al-Ishaq R.K., Liskova A., Kubatka P., Büsselberg D. (2021). Enzymatic Metabolism of Flavonoids by Gut Microbiota and Its Impact on Gastrointestinal Cancer. Cancers.

[23] Boyle S.P., Dobson V.L., Duthie S.J., Hinselwood D.C., Kyle J.A., Collins A.R. (2000). Bioavailability and efficiency of rutin as an antioxidant: A human supplementation study. Eur. J. Clin. Nutr..

[24] Malekpour M., Ebrahiminezhad A., Karimi Z., Saadi M.I., Berenjian A. (2025). Current strategies for rutin nano-formulation; a promising bioactive compound with increased efficacy. Bioprocess Biosyst. Eng..

[25] Nautiyal G., Minocha N., Sharma S.K., Yadav K., Kaushik D., Pandey P. (2025). Nano-Rutin: A Promising Solution for Alleviating Various Disorders. Recent Pat. Nanotechnol..

[26] de Kok M., Schaapherder A., Bloeme-Ter Horst J.R., Faro M.L.L., de Vries D.K., Ploeg R.J., Bakker J.A., Lindeman J.H.N. (2025). Clinical ischemia-reperfusion injury: Driven by reductive rather than oxidative stress? A narrative review. Biochim. Biophys. Acta Bioenerg..

[27] Bertero E., Popoiu T.A., Maack C. (2024). Mitochondrial calcium in cardiac ischemia/reperfusion injury and cardioprotection. Basic Res. Cardiol..

[28] Lian Y.Q., Li P.F., Guo Y., Tao Y.L., Liu Y.N., Liang Z.Y., Zhu S.F. (2024). Interaction between ischemia-reperfusion injury and intestinal microecology in organ transplantation and its therapeutic prospects. Front. Immunol..

[29] Li W., Liao Y., Chen J., Kang W., Wang X., Zhai X., Xue Y., Zhang W., Xia Y., Cui D. (2025). Ischemia-Reperfusion injury: A roadmap to precision therapies. Mol. Asp. Med..

[30] Carlström M., Rannier Ribeiro Antonino Carvalho L., Guimaraes D., Boeder A., Schiffer T.A. (2024). Dimethyl malonate preserves renal and mitochondrial functions following ischemia-reperfusion via inhibition of succinate dehydrogenase. Redox Biol..

[31] Bei Y., Zhu Y., Zhou J., Ai S., Yao J., Yin M., Hu M., Qi W., Spanos M., Li L. (2024). Inhibition of Hmbox1 Promotes Cardiomyocyte Survival and Glucose Metabolism Through Gck Activation in Ischemia/Reperfusion Injury. Circulation.

[32] Wu J., Gao P., Yang C., Zhuang F., Luo Y., Wen F., Zhang P., Wang L., Xie H., Dai C. (2025). Targeting mitochondrial complex I of CD177(+) neutrophils alleviates lung ischemia-reperfusion injury. Cell Rep. Med..

[33] Mohammad A., Babiker F., Al-Bader M. (2023). Effects of Apocynin, a NADPH Oxidase Inhibitor, in the Protection of the Heart from Ischemia/Reperfusion Injury. Pharmaceuticals.

[34] Li H., Wang X., Deng D., Lv S., Huang L., Wang X. (2025). Advances in understanding the role of mitochondria in renal ischemia-reperfusion injury. Clin. Exp. Nephrol..

[35] Song Z., Xia Y., Shi L., Zha H., Huang J., Xiang X., Li H., Huang H., Yue R., Wang H. (2024). Inhibition of Drp1- Fis1 interaction alleviates aberrant mitochondrial fragmentation and acute kidney injury. Cell Mol. Biol. Lett..

[36] Yu L., Wang X., Lei Q., Liu Y., Li Z., Dai X., Song Z., He Y., Gao S., Yu C. (2025). Tongmai Yangxin pill alleviates myocardial ischemia/reperfusion injury by regulating mitochondrial fusion and fission through the estrogen receptor alpha/peroxisome proliferator-activated receptor gamma coactivator-1 alpha signaling pathway. J. Ethnopharmacol..

[37] Li X.T., Li X.Y., Tian T., Yang W.H., Lyv S.G., Cheng Y., Su K., Lu X.H., Jin M., Xue F.S. (2025). The UCP2/PINK1/LC3b-mediated mitophagy is involved in the protection of NRG1 against myocardial ischemia/reperfusion injury. Redox Biol..

[38] Zhang Y., Wang Z., Jia C., Yu W., Li X., Xia N., Nie H., Wikana L.P., Chen M., Ni Y. (2024). Blockade of Hepatocyte PCSK9 Ameliorates Hepatic Ischemia-Reperfusion Injury by Promoting Pink1-Parkin-Mediated Mitophagy. Cell Mol. Gastroenterol. Hepatol..

[39] George J., Lu Y., Tsuchishima M., Tsutsumi M. (2024). Cellular and molecular mechanisms of hepatic ischemia-reperfusion injury: The role of oxidative stress and therapeutic approaches. Redox Biol..

[40] Xiang Q., Yi X., Zhu X.H., Wei X., Jiang D.S. (2024). Regulated cell death in myocardial ischemia-reperfusion injury. Trends Endocrinol. Metab..

[41] Chen L., Mao L.S., Xue J.Y., Jian Y.H., Deng Z.W., Mazhar M., Zou Y., Liu P., Chen M.T., Luo G. (2024). Myocardial ischemia-reperfusion injury: The balance mechanism between mitophagy and NLRP3 inflammasome. Life Sci..

[42] Liu J., Luo R., Zhang Y., Li X. (2024). Current status and perspective on molecular targets and therapeutic intervention strategy in hepatic ischemia-reperfusion injury. Clin. Mol. Hepatol..

[43] Leng J., Cao Z., Li L., Hu D., Luo Y., Tu B., Cao X., Tao R., Jiang Y., Tie H. (2025). PGC1α alleviates M1 macrophage polarization through dual regulation of succinate metabolism and TRAF5 expression to mitigate TLR4/NF-κB-driven inflammatory cascades and myocardial ischemia/reperfusion injury. Inflamm. Res..

[44] Li J., Dong S., Quan S., Ding S., Zhou X., Yu Y., Wu Y., Huang W., Shi Q., Li Q. (2024). Nuciferine reduces inflammation induced by cerebral ischemia-reperfusion injury through the PI3K/Akt/NF-κB pathway. Phytomedicine.

[45] Weiss A., Ding Y. (2026). Beyond Reperfusion: Adjunctive Therapies Targeting Inflammation, Edema, and Blood-Brain Barrier Dysfunction in Ischemic Stroke. Cerebrovasc Dis..

[46] Wang J., Xiong M., Fan Y., Liu C., Wang Q., Yang D., Yuan Y., Huang Y., Wang S., Zhang Y. (2022). Mecp2 protects kidney from ischemia-reperfusion injury through transcriptional repressing IL-6/STAT3 signaling. Theranostics.

[47] Lan R., Zhang Y., Fu X.Q., Lan R., Zhang Y., Fu X.Q., Wang M.M., Zou X.H., Wang W.W., Shen X.M. (2025). Xiao-xu-ming decoction improved synaptic damage after acute cerebral ischemia and reperfusion via JAK2/STAT3 pathway in rats. J. Ethnopharmacol..

[48] Prionas A., Hamaoui K., Vanezis K., Reebye V., Habib N., Papalois V. (2024). The Effect of Interleukin-10 Immunotherapy on Renal Ischemia-Reperfusion Injury: A Systematic Review and Meta-Analysis of Preclinical Studies. Int. J. Mol. Sci..

[49] Li Q., Nie H. (2024). Advances in lung ischemia/reperfusion injury: Unraveling the role of innate immunity. Inflamm. Res..

[50] Lang X., Zhong C., Su L., Qin M., Xie Y., Shan D., Cui Y., Shi M., Li M., Quan H. (2024). Edgeworthia gardneri (Wall.) Meisn. Ethanolic Extract Attenuates Endothelial Activation and Alleviates Cardiac Ischemia-Reperfusion Injury. Molecules.

[51] Tonnus W., Locke S., Meyer C., Maremonti F., Eggert L., von Mässenhausen A., Bornstein S.R., Green D.R., Linkermann A. (2022). Rubicon-deficiency sensitizes mice to mixed lineage kinase domain-like (MLKL)-mediated kidney ischemia-reperfusion injury. Cell Death Dis..

[52] Ye B., Xu D., Zhong L., Wang Y., Wang W., Xu H., Han X., Min J., Wu G., Huang W. (2025). Ubiquitin-specific protease 25 improves myocardial ischemia-reperfusion injury by deubiquitinating NLRP3 and negatively regulating NLRP3 inflammasome activity in cardiomyocytes. Clin. Transl. Med..

[53] Zhang M., Liu Q., Meng H., Duan H., Liu X., Wu J., Gao F., Wang S., Tan R., Yuan J. (2024). Ischemia-reperfusion injury: Molecular mechanisms and therapeutic targets. Signal Transduct. Target. Ther..

[54] Lu C., Xu C., Li S., Ni H., Yang J. (2025). Liraglutide and GLP-1(9-37) alleviated hepatic ischemia-reperfusion injury by inhibiting ferroptosis via GSK3β/Nrf2 pathway and SMAD159/Hepcidin/FTH pathway. Redox Biol..

[55] Kang P., Zhou X., Zhao S., Yu W., Ye Z., Cheng F. (2025). Inhibitors of p53 Apoptosis-Stimulating Protein Mitigate Acute Kidney Injury by Modulating the HIF-1α/SLC7A11 Pathway to Suppress Ferroptosis. J. Cell Mol. Med..

[56] Hu F., Zhang S., Chen X., Fu X., Guo S., Jiang Z., Chen K. (2020). MiR-219a-2 relieves myocardial ischemia-reperfusion injury by reducing calcium overload and cell apoptosis through HIF1α/ NMDAR pathway. Exp. Cell Res..

[57] Zhao X., Li Q., Zhu X., Jiao Y., Yang H., Feng J. (2025). Protein modifications in hepatic ischemia-reperfusion injury: Molecular mechanisms and targeted therapy. Front. Immunol..

[58] Wang L., Li D., Yao F., Feng S., Tong C., Rao R., Zhong M., Wang X., Feng W., Hu Z. (2025). Serpina3k lactylation protects from cardiac ischemia reperfusion injury. Nat. Commun..

[59] Zhou J., Zhang J., Xu F., Gao H., Wang L., Zhao Y., Li K. (2024). AST-120 alleviates renal ischemia-reperfusion injury by inhibiting HK2-mediated glycolysis. Mol. Med..

[60] Lee T.L., Shen W.C., Chen Y.C., Lai T.C., Lin S.R., Lin S.W., Yu I.S., Yeh Y.H., Li T.K., Lee I.T. (2025). Mir221- and Mir222-enriched adsc-exosomes mitigate PM exposure-exacerbated cardiac ischemia-reperfusion injury through the modulation of the BNIP3-MAP1LC3B-BBC3/PUMA pathway. Autophagy.

[61] Yin L., Li L., Gao M., Qi Y., Xu L., Peng J. (2024). circMIRIAF aggravates myocardial ischemia-reperfusion injury via targeting miR-544/WDR12 axis. Redox Biol..

[62] Du Z.W., Li Y.S., Jiang X.C., Gao J.Q. (2025). Nanoparticles Designed Based on the Blood-Brain Barrier for the Treatment of Cerebral Ischemia-Reperfusion Injury. Small.

[63] Wang S., Shi X., Xiong T., Chen Q., Yang Y., Chen W., Zhang K., Nan Y., Huang Q., Ai K. (2024). Inhibiting Mitochondrial Damage for Efficient Treatment of Cerebral Ischemia-Reperfusion Injury Through Sequential Targeting Nanomedicine of Neuronal Mitochondria in Affected Brain Tissue. Adv. Mater..

[64] Ortolani O., Caggiano M., Mannelli R., Gogliettino A., Tufano R. (1995). Protection from ischemia-reperfusion damage in patients with stroke: The role of rutin and GSH. Transplant. Proc..

[65] Pu F., Mishima K., Irie K., Motohashi K., Tanaka Y., Orito K., Egawa T., Kitamura Y., Egashira N., Iwasaki K. (2007). Neuroprotective effects of quercetin and rutin on spatial memory impairment in an 8-arm radial maze task and neuronal death induced by repeated cerebral ischemia in rats. J. Pharmacol. Sci..

[66] Khan M.M., Ahmad A., Ishrat T., Khuwaja G., Srivastawa P., Khan M.B., Raza S.S., Javed H., Vaibhav K., Khan A. (2009). Rutin protects the neural damage induced by transient focal ischemia in rats. Brain Res..

[67] Abd-El-Fattah A.A., El-Sawalhi M.M., Rashed E.R., El-Ghazaly M.A. (2010). Possible role of vitamin E, coenzyme Q10 and rutin in protection against cerebral ischemia/reperfusion injury in irradiated rats. Int. J. Radiat. Biol..

[68] Annapurna A., Ansari M.A., Manjunath P.M. (2013). Partial role of multiple pathways in infarct size limiting effect of quercetin and rutin against cerebral ischemia-reperfusion injury in rats. Eur. Rev. Med. Pharmacol. Sci..

[69] Jang J.W., Lee J.K., Hur H., Kim T.W., Joo S.P., Piao M.S. (2014). Rutin improves functional outcome via reducing the elevated matrix metalloproteinase-9 level in a photothrombotic focal ischemic model of rats. J. Neurol. Sci..

[70] Hao G., Dong Y., Huo R., Wen K., Zhang Y., Liang G. (2016). Rutin Inhibits Neuroinflammation and Provides Neuroprotection in an Experimental Rat Model of Subarachnoid Hemorrhage, Possibly Through Suppressing the RAGE-NF-κB Inflammatory Signaling Pathway. Neurochem. Res..

[71] Zhào H., Liu Y., Zeng J., Li D., Zhang W., Huang Y. (2018). Troxerutin and Cerebroprotein Hydrolysate Injection Protects Neurovascular Units from Oxygen-Glucose Deprivation and Reoxygenation-Induced Injury In Vitro. Evid.-Based Complement. Altern. Med..

[72] Ma W., Wang S., Liu X., Tang F., Zhao P., Cheng K., Zheng Q., Zhuo Y., Zhao X., Li X. (2019). Protective effect of troxerutin and cerebroprotein hydrolysate injection on cerebral ischemia through inhibition of oxidative stress and promotion of angiogenesis in rats. Mol. Med. Rep..

[73] Sui R., Zang L., Bai Y. (2019). Administration of troxerutin and cerebroprotein hydrolysate injection alleviates cerebral ischemia/reperfusion injury by down-regulating caspase molecules. Neuropsychiatr. Dis. Treat..

[74] Zhào H., Wang R., Zhang Y., Liu Y., Huang Y. (2021). Neuroprotective effects of troxerutin and cerebroprotein hydrolysate injection on the neurovascular unit in a rat model of Middle cerebral artery occlusion. Int. J. Neurosci..

[75] Nassiri-Asl M., Ghorbani A., Salehisar S., Asadpour E., Sadeghnia H.R. (2020). Effect of rutin on oxidative DNA damage in PC12 neurons cultured in nutrients deprivation condition. Iran. J. Basic Med. Sci..

[76] Gao C., Song Y., Dou T., Jiang S., Wu H., Seshadri V.D., Veeraraghavan V.P., Hou P. (2021). Troxerutin Abrogates Ischemic/Reperfusion-Induced Brain Injury through Ameliorating Oxidative Stress and Neuronal Inflammation by Inhibiting the Expression of NLRP3 in Sprague Dawley Rats. J. Environ. Pathol. Toxicol. Oncol..

[77] Zhao T., He F., Zhao K., Yuxia L., Li H., Liu X., Cen J., Duan S. (2023). A Triple-Targeted Rutin-Based Self-Assembled Delivery Vector for Treating Ischemic Stroke by Vascular Normalization and Anti-Inflammation via ACE2/Ang1-7 Signaling. ACS Cent. Sci..

[78] Rana A.K., Kumar R., Shukla D.N., Singh D. (2023). Lithium co-administration with rutin improves post-stroke neurological outcomes via suppressing Gsk-3β activity in a rat model. Free Radic. Biol. Med..

[79] Wang Y., Shou X., Fan Z., Cui J., Xue D., Wu Y. (2022). A Systematic Review and Meta-Analysis of Phytoestrogen Protects Against Myocardial Ischemia/Reperfusion Injury: Pre-Clinical Evidence From Small Animal Studies. Front. Pharmacol..

[80] Blasig I.E., Löwe H., Ebert B. (1987). Radical trapping and lipid peroxidation during myocardial reperfusion injury—Radical scavenging by troxerutin in comparison to mannitol. Biomed. Biochim. Acta.

[81] Ali M.S., Mudagal M.P., Goli D. (2009). Cardioprotective effect of tetrahydrocurcumin and rutin on lipid peroxides and antioxidants in experimentally induced myocardial infarction in rats. Pharmazie.

[82] Jeong J.J., Ha Y.M., Jin Y.C., Lee E.J., Kim J.S., Kim H.J., Seo H.G., Lee J.H., Kang S.S., Kim Y.S. (2009). Rutin from Lonicera japonica inhibits myocardial ischemia/reperfusion-induced apoptosis in vivo and protects H9c2 cells against hydrogen peroxide-mediated injury via ERK1/2 and PI3K/Akt signals in vitro. Food Chem. Toxicol..

[83] Challa S.R., Akula A., Metla S., Gopal P.N. (2011). Partial role of nitric oxide in infarct size limiting effect of quercetin and rutin against ischemia-reperfusion injury in normal and diabetic rats. Indian J. Exp. Biol..

[84] Bhandary B., Piao C.S., Kim D.S., Lee G.H., Chae S.W., Kim H.R., Chae H.J. (2012). The protective effect of rutin against ischemia/reperfusion-associated hemodynamic alteration through antioxidant activity. Arch. Pharm. Res..

[85] Mokhtari B., Badalzadeh R., Alihemmati A., Mohammadi M. (2015). Phosphorylation of GSK-3β and reduction of apoptosis as targets of troxerutin effect on reperfusion injury of diabetic myocardium. Eur. J. Pharmacol..

[86] Singh H., Kaur P., Kaur P., Muthuraman A., Singh G., Kaur M. (2015). Investigation of therapeutic potential and molecular mechanism of vitamin P and digoxin in I/R-induced myocardial infarction in rat. Naunyn-Schmiedeberg’s Arch. Pharmacol..

[87] Shu L., Zhang W., Huang C., Huang G., Su G. (2017). Troxerutin Protects Against Myocardial Ischemia/Reperfusion Injury via Pi3k/Akt Pathway in Rats. Cell Physiol. Biochem..

[88] Badalzadeh R., Baradaran B., Alihemmati A., Yousefi B., Abbaszadeh A. (2017). Troxerutin Preconditioning and Ischemic Postconditioning Modulate Inflammatory Response after Myocardial Ischemia/Reperfusion Injury in Rat Model. Inflammation.

[89] Najafi M., Noroozi E., Javadi A., Badalzadeh R. (2018). Anti-arrhythmogenic and anti-inflammatory effects of troxerutin in ischemia/reperfusion injury of diabetic myocardium. Biomed. Pharmacother..

[90] Shu L., Zhang W., Huang G., Huang C., Zhu X., Su G., Xu J. (2019). Troxerutin attenuates myocardial cell apoptosis following myocardial ischemia-reperfusion injury through inhibition of miR-146a-5p expression. J. Cell Physiol..

[91] Yang H., Wang C., Zhang L., Lv J., Ni H. (2019). Rutin alleviates hypoxia/reoxygenation-induced injury in myocardial cells by up-regulating SIRT1 expression. Chem.-Biol. Interact..

[92] Yu Z.P., Yu H.Q., Li J., Li C., Hua X., Sheng X.S. (2020). Troxerutin attenuates oxygen-glucose deprivation and reoxygenation-induced oxidative stress and inflammation by enhancing the PI3K/AKT/HIF-1α signaling pathway in H9C2 cardiomyocytes. Mol. Med. Rep..

[93] Wang Q., Zhang Y., Lu R., Zhao Q., Gao Y. (2024). The multiple mechanisms and therapeutic significance of rutin in metabolic dysfunction-associated fatty liver disease (MAFLD). Fitoterapia.

[94] Acquaviva R., Lanteri R., Li Destri G., Caltabiano R., Vanella L., Lanzafame S., Di Cataldo A., Li Volti G., Di Giacomo C. (2009). Beneficial effects of rutin and L-arginine coadministration in a rat model of liver ischemia-reperfusion injury. Am. J. Physiol. Gastrointest. Liver Physiol..

[95] Olmez H., Tosun M., Unver E., Cimen F.K., Arslan Y.K., Gulaboglu M., Suleyman B. (2023). The role of polymorphonuclear leukocytes in distant organ (lung) oxidative damage of liver ischemia/reperfusion and the protective effect of rutin. Adv. Clin. Exp. Med..

[96] da Silva Rangel R., Tamiosso R.T., da Silva R.S., da Silva de Oliveira L.S., Biscarra Bortolotto Paz M.F., De Paula Martins T.T., Mazaro A., de Moraes Chitolina A.B., Maurente M.M., de Andrade C.M. (2025). Rutin attenuates oxidative damage-induced renal injury in rats experimentally infected with Cryptococcus neoformans by improving antioxidant capacity and reducing fungal burden. Microb. Pathog..

[97] Shan Q., Zheng G.H., Han X.R., Wen X., Wang S., Li M.Q., Zhuang J., Zhang Z.F., Hu B., Zhang Y. (2018). Troxerutin Protects Kidney Tissue against BDE-47-Induced Inflammatory Damage through CXCR4-TXNIP/NLRP3 Signaling. Oxid. Med. Cell Longev..

[98] Wang B., Liu D., Zhu Q.H., Li M., Chen H., Guo Y., Fan L.P., Yue L.S., Li L.Y., Zhao M. (2016). Rutin ameliorates kidney interstitial fibrosis in rats with obstructive nephropathy. Int. Immunopharmacol..

[99] Korkmaz A., Kolankaya D. (2010). Protective effect of rutin on the ischemia/reperfusion induced damage in rat kidney. J. Surg. Res..

[100] Korkmaz A., Kolankaya D. (2013). Inhibiting inducible nitric oxide synthase with rutin reduces renal ischemia/reperfusion injury. Can. J. Surg..

[101] Muthuraman A., Kaur P., Kaur P., Singh H., Boparai P.S. (2015). Ameliorative potential of vitamin P and digoxin in ischemic-reperfusion induced renal injury using the Langendorff apparatus. Life Sci..

[102] Feng W., Zhu N., Xia Y., Huang Z., Hu J., Guo Z., Li Y., Zhou S., Liu Y., Liu D. (2024). Melanin-like nanoparticles alleviate ischemia-reperfusion injury in the kidney by scavenging reactive oxygen species and inhibiting ferroptosis. iScience.

[103] Wei S.M., Yan Z.Z., Zhou J. (2011). Protective effect of rutin on testicular ischemia-reperfusion injury. J. Pediatr. Surg..

[104] Rotimi D.E., Elebiyo T.C., Ojo O.A. (2023). Therapeutic potential of rutin in male infertility: A mini review. J. Integr. Med..

[105] Akondi B.R., Challa S.R., Akula A. (2011). Protective effects of rutin and naringin in testicular ischemia-reperfusion induced oxidative stress in rats. J. Reprod. Infertil..

[106] Sirotkin A.V. (2024). Positive effects of rutin on female reproduction. Reprod. Domest. Anim..

[107] Nayki C., Nayki U., Keskin Cimen F., Kulhan M., Yapca O.E., Kurt N., Bilgin Ozbek A. (2018). The effect of rutin on ovarian ischemia-reperfusion injury in a rat model. Gynecol. Endocrinol..

[108] Ren Y., Wang L., Wang D., Huang J., Wang O., Ding G. (2025). Rutin-Whey Protein Nanoparticles Inhibit D-Galactose-Induced Skeletal Muscle Dysfunction by Modulating Gut Microbiota and Metabolic Pathways. Nutrients.

[109] Liu Y., Gou L., Fu X., Li S., Lan N., Yin X. (2013). Protective effect of rutin against acute gastric mucosal lesions induced by ischemia-reperfusion. Pharm. Biol..

[110] Zhang Y., Xiong W., Ren Y., Huang J., Wang X., Wang O., Cai S. (2024). Preparation of Rutin-Whey Protein Pickering Emulsion and Its Effect on Zebrafish Skeletal Muscle Movement Ability. Nutrients.

[111] Moldovan M., Muntean M., Schauer S.A., Moldovan R., Mitrea D.R. (2025). Oxidative Stress and Ultrastructural Analysis in Heart, Aorta, Skeletal Muscle and Lung of Rats Treated with N-Acetylcysteine or Rutin After Sprint Running. J. Funct. Morphol. Kinesiol..

[112] Seo S., Lee M.S., Chang E., Shin Y., Oh S., Kim I.H., Kim Y. (2015). Rutin Increases Muscle Mitochondrial Biogenesis with AMPK Activation in High-Fat Diet-Induced Obese Rats. Nutrients.

[113] Neumayer C., Fügl A., Nanobashvili J., Blumer R., Punz A., Gruber H., Polterauer P., Huk I. (2006). Combined enzymatic and antioxidative treatment reduces ischemia-reperfusion injury in rabbit skeletal muscle. J. Surg. Res..

[114] Su A.L., Zhao S., Zhu H.N., Qiao Y., Zhang T. (2024). Rutin promotes M2 phenotype microglia polarization by suppressing the JAK/STAT3 signaling to protect against retinal ischemia-reperfusion injury. Biomed. Res..

